# Phylogeography of *Partamona rustica* (Hymenoptera, Apidae), an Endemic Stingless Bee from the Neotropical Dry Forest Diagonal

**DOI:** 10.1371/journal.pone.0164441

**Published:** 2016-10-10

**Authors:** Elder Assis Miranda, Henrique Batalha-Filho, Carlos Congrains, Antônio Freire Carvalho, Kátia Maria Ferreira, Marco Antonio Del Lama

**Affiliations:** 1 Departamento de Genética e Evolução, Universidade Federal de São Carlos, São Carlos, São Paulo, Brazil; 2 Departamento de Ciências Biológicas, Universidade Estadual de Santa Cruz, Ilhéus, Bahia, Brazil; 3 Departamento de Zoologia, Instituto de Biologia, Universidade Federal da Bahia, Salvador, Bahia, Brazil; Universidade de São paulo, BRAZIL

## Abstract

The South America encompasses the highest levels of biodiversity found anywhere in the world and its rich biota is distributed among many different biogeographical regions. However, many regions of South America are still poorly studied, including its xeric environments, such as the threatened *Caatinga* and *Cerrado* phytogeographical domains. In particular, the effects of Quaternary climatic events on the demography of endemic species from xeric habitats are poorly understood. The present study uses an integrative approach to reconstruct the evolutionary history of *Partamona rustica*, an endemic stingless bee from dry forest diagonal in Brazil, in a spatial-temporal framework. In this sense, we sequenced four mitochondrial genes and genotyped eight microsatellite loci. Our results identified two population groups: one to the west and the other to the east of the São Francisco River Valley (SFRV). These groups split in the late Pleistocene, and the Approximate Bayesian Computation approach and phylogenetic reconstruction indicated that *P*. *rustica* originated in the west of the SFRV, subsequently colonising eastern region. Our tests of migration detected reduced gene flow between these groups. Finally, our results also indicated that the inferences both from the genetic data analyses and from the spatial distribution modelling are compatible with historical demographic stability.

## Introduction

South America encompasses the highest levels of biodiversity found anywhere in the world [1] and its rich biota is distributed among many different biogeographical regions [2]. Studies of the diversification of the Neotropical biota have revealed a complex evolutionary history [3] that appears to have fluctuated continuously throughout the Tertiary and Quaternary [4]. This diversification was influenced by tectonic [5] and paleoclimatic events [6, 7]. Nevertheless, many regions of South America are still poorly studied, including its xeric environments [8, 3], such as the threatened *Caatinga* and *Cerrado* phytogeographical domains. In particular, the effects of Quaternary climatic events on the demography of endemic species from xeric habitats are poorly understood.

Phylogeographic studies have indicated that Pleistocene climatic changes may have influenced the species richness, spatial distribution, endemism, genetic diversity, historical demography and biogeographic patterns of the Neotropical biota [9, 10]. Present-day Neotropical biodiversity is frequently explained by the Pleistocene “refugia” hypothesis, which relates successive climatic-vegetation cycles during the Pleistocene, in particular glacial events, to vicariant processes and the expansion or retraction of species ranges [11, 12]. In this sense, three main refuges between the Last Glacial Maximum (~21 ka) and the present day through the Seasonally Dry Tropical Forests (SDTF) of South America—the *Caatinga*, Misiones/Piemonte and Chiquitano refuges—were proposed [13]. The *Caatinga* refuge, which covers most of the present-day distribution of its area, is the largest stable area of SDTF. However, some *Caatinga* ecoregions have weak climatic stability [13]. Previous study has investigated the historical distribution of the *Cerrado* and found evidence of two savanna corridors and predicted the presence of a large refuge in the north-eastern extreme of this area [14].

The geological and paleo-climatic history of the *Caatinga* and *Cerrado* is complex and controversial [8], and understanding the evolutionary history of the animal groups adapted to these semiarid regions can provide important insights into the role of historical events in the diversification of their endemic biota [14, 10]. To shed light on this biogeographic history, we investigated the role of Pleistocene climatic changes on the biota of the Brazilian *Caatinga* and *Cerrado* using the stingless bee *Partamona rustica* [15] as a model. This bee is endemic to northeastern Brazil, where it inhabits the *Caatinga* of southwestern state of Bahia and areas between the *Caatinga* and the *Cerrado* in northern state of Minas Gerais [15]. Its nests are often associated with those of the arboreal termite *Constrictotermes cyphergaster*, where they build its nests [15, 16]. This bee is a floral visitor of at least 62 plants, and is probably an important pollinator in these areas. Because of increasing anthropogenic impacts, this species is now rare in some areas [16].

In the present study, we used an integrative approach to reconstruct the evolutionary history of *P*. *rustica* in a spatial-temporal framework. Specifically, we investigated the genetic diversity and structure of its populations, and how climatic changes during the late Pleistocene may have influenced the demographic history of its populations. We also inferred species distribution patterns since the late Pleistocene to identify possible species-specific refugia. We then applied a coalescent-based Approximate Bayesian Computation (ABC) approach to test competing scenarios of the origin and dispersal of *P*. *rustica* populations across the species current geographic range.

## Materials and Methods

### Sampling and Laboratory Procedures

**Fig 1:**
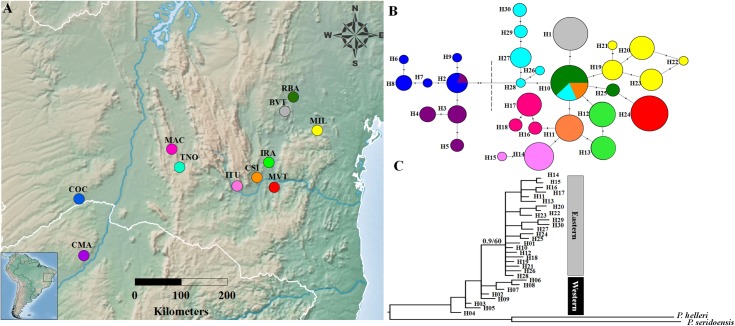
**Map of the geographical distribution of populations (A), median-joining haplotype network (B) and phylogenetic reconstruction by Bayesian inference (C) based on four concatenated mtDNA genes in *P*. *rustica***. The coloured circles in the map represent the 11 localities sampled in the present study (listed in Table 1). The haplotypes are coloured according to the scheme in “A”. The dotted line in the haplotype network separates the eastern and western groups. The black vertical dashes represent the positions of the mutations in the haplotypes. In “C”, numbers above branches indicate support values expressed at Bayesian posterior probabilities / Maximum likelihood bootstrap.

We sampled adult *P*. *rustica* workers from 145 nests from 11 localities in the *Caatinga* of south-western Bahia (localities 1 to 9 in Table 1) and in the transition zone between the *Caatinga* and *Cerrado* in northern Minas Gerais (localities 10 and 11 in Table 1), in Brazil (Fig 1A), between May 2012 and January 2014. All specimens were preserved in ethanol prior to the molecular analyses and vouchers are deposited in the Laboratório de Genética Evolutiva de Himenópteros (LGEH) at the Universidade Federal de São Carlos and in the Entomological Camargo Collection at the Universidade de São Paulo (FFCLRP—USP) (in Brazil). All necessary research permits for fieldwork and collection of samples by EAM were issued by the Brazilian Institute for Biodiversity Conservation (Instituto Chico Mendes de Conservação da Biodiversidade—ICMBio) recorded by SISBio (permit number 31750). Field studies did not involve endangered or protected species.

**Table 1:** Localities sampled in the present study, geographic coordinates (S and W), altitude (A, in meters), number of nests (N). All sites are located in the Brazilian state of Bahia, except Cônego Marinho, in the state of Minas Gerais.

	Localities	Code	S	W	A	N
1	Boa Vista do Tupim	BVT	-12.502	-40.471	328	14
2	Contendas do Sincorá	CSI	-13.794	-41.017	300	12
3	Iramaia	IRA	-13.862	-40.081	382	15
4	Ituaçu	ITU	-13.882	-41.325	586	11
5	Macaúbas	MAC	-12.243	-40.250	756	11
6	Milagres	MIL	-12.931	-39.720	365	20
7	Manoel Vitorino	MVT	-13.939	-40.563	263	16
8	Ruy Barbosa	RBA	-12.228	-40.311	314	12
9	Tanque Novo	TNO	-13.595	-42.521	789	14
10	Cocos	COC	-14.158	-44.403	587	10
11	Cônego Marinho	CMA	-15.314	-44.382	632	10
					Total	**145**

Total DNA was extracted from one worker per colony using Sheppard and McPheron’s protocol [17]. The mitochondrial genes 12S, 16S, COI and the terminal region of subunit I and beginning of cytochrome oxidase subunit II (COI-COII) were amplified partially using the techniques described in S1 Text. Information on the sequence of the primers and the fragment size can be found in S1 Table. All mitochondrial haplotypes have been deposited in GenBank (accession numbers KT765104-KT765129). In addition, we genotyped eight microsatellite loci (SSR) for *P*. *rustica*. Six of these loci were described for *Partamona helleri* (Phel1, Phel2, Phel3, Phel4, Phel6 and Phel7) [18] and two for *Melipona bicolor* (Mbi 232 and Mbi 254) [19]. The PCR cycling conditions of these loci followed the protocol described in other study [18]. Information on the sequence of the primers, repeat motifs, annealing temperatures and the respective fluorophores are available in S2 Table.

### Genetic Diversity

The electropherograms were edited and combined in contigs using the Codon Code Aligner v3.7.1 software (CodonCode, Dedham, Massachusetts, United States). The sequences were aligned using CLUSTAL W in the BioEdit 7.0.9.0 program [20]. All alignments were inspected and corrected visually.

To assess genetic diversity in *P*. *rustica*, we estimated the number of variable sites (S), number of haplotypes (h), haplotype diversity (Hd) and nucleotide diversity (π) for each mitochondrial gene region and for the concatenated gene regions using DnaSP v5.10.01 [21].

For the analyses of the SSR dataset, Microchecker v. 2.2.3 [22] was first used to check for null alleles, scoring errors and large allele dropouts. We estimated the number of alleles, allelic richness (Ar—mean number of alleles, corrected by the smallest sample number) and genetic diversity using Fstat 2.9.3.2 [23]. We also applied Fisher’s exact test to test the microsatellite loci for deviations from Hardy-Weinberg equilibrium (HWE) and linkage disequilibrium, using the Markov chain approach in Genepop v.4.2 [24].

### Tests of Population Structure

We obtained a haplotype network for the concatenated mitochondrial gene regions using the median-joining network method [25] implemented in NETWORK 4.6.1.3 (http://www.fluxus-engineering.com/). In order to verify the occurrence of population groups on the SSR dataset, we used the AMOVA-based K-means clustering method [26] in kMeans v.1.1. (http://www.patrickmeirmans.com/software/). We also conducted a spatial analysis of molecular variance (SAMOVA) using the SSR and mtDNA (all concatenated data) datasets separately in SAMOVA 2.0 [27]. For further information, see S1 Text.

We also implemented the Mantel test using the Isolation by Distance Web Service 3.23 (IBDWS) [28] to investigate the potential correlation between genetic (pairwise Φ_ST_) and geographic (in kilometres) distances, considering all populations. The significance of this analysis was obtained with 10,000 permutations of both mtDNA (all concatenated data) and SSR datasets, in analyses run separately.

### Phylogenetic Analyses

We implemented Bayesian inference (BI) and maximum likelihood (ML) to estimate the phylogenetic between *P*. *rustica* lineages based on haplotype of four concatenated mitochondrial regions. The best fit model was selected using MrModeltest v2 [29] based on the Akaike information criterion (AIC), assuming four partitions (COI, COII, 12S and 16S). We used two independent Bayesian runs of 20 million generations with four chains of Markov chain Monte Carlo (MCMC) for each. First two thousand generations were discarded as burn-in, after which trees were sampled every 1,000 generations. This analysis was performed in MrBayes v3.2.2 program [30]. Chain convergence was checked using the likelihood plots for each run using Tracer 1.6 (http://beast.bio.ed.ac.uk/Tracer) and we accepted the results if ESS values were >200. On the other hand, the ML inference was run under the GTRAC model; invariable sites and gamma distribution were estimated independently for each partition during the run. Node supports of the maximum likelihood analysis were estimated with 1,000 bootstrap replications. This analysis was performed in RAxML program [31]. For both the phylogenetic analyses we used samples of *Partamona helleri* and *Partamona seridoensis* as outgroup taxa. Tree obtained for each inference was visualized and edited using FigTree 1.4 (http://tree.bio.ed.ac.uk/software/figtree/).

### Population Demography, Divergence Times and Migration

To evaluate the population size dynamics over time we implemented the Bayesian Skyline Plot (BSP) for each population group using BEAST 1.8.1 [32], based on mtDNA concatenated COI and COI-COII regions. We implemented two independent runs with the following parameters: a strict clock, 60 million generations for the western group and 200 million generations for the eastern group, sampling parameters at every 1000 generations of the Markov Chain Monte Carlo (MCMC) analysis, and 10% as burn-in period. To obtain the absolute times we used the COI substitution rate of 1.3–1.9% per lineage per million years (with a uniform distributed prior) calibrated for other hymenopterans [33, 34]. We used the HKY substitution model for the western group and the GTR+I model for the eastern group, as selected by jModeltest v.2.1.5 [35], based on the Akaike information criterion (AIC). We checked for convergence between runs and the performance of the analysis using Tracer 1.5 [36], and accepted the results if effective sample size (ESS) values were greater than 200.

Divergence times, effective population sizes and migration rates between western and eastern *P*. *rustica* groups were calculated based on the isolation with migration (IM) model [37, 38] using IMa2 [39], for the COI and COI-COII sequences. This program estimated six demographic parameters for pairs of populations: the splitting time (t = mutation-scaled time since divergence), migration rates between groups (m_west>east_ and m_east>west_ = mutation-scaled migration rate), and the effective population sizes of the eastern, western and ancestral populations (θ_eastern_, θ_western_ and θ_ancestral_).

We first conducted series of relatively short preliminary runs in the ‘MCMC mode’. These short runs were used to adjust the run duration and prior distribution for each parameter and the heating scheme for the subsequent, longer runs. For the final run, we used 40 MCMC chains with a geometric heating scheme (g1 = 0.975 and g2 = 0.75), with the maximum prior population sizes set at 30, migration rates at 20 and divergence time at 20. We used the HKY substitution model and an inheritance scalar of 0.25. We assumed the same mutation rate for COI (1.3–1.9% per lineage per million years) as in BEAST and a generation time of one year. The run had eight million steps. The first two million steps were discarded as burn-in. We also checked convergence by analysing the posterior distributions, and the ESS values that were higher than 200. We also tested the SSR dataset (on its own or together with the mtDNA data). We removed the ribosomal regions (12S and 16S) from the IMa2 and BSP analyses because no substitution rates have yet been estimated for these regions in the Hymenoptera. In all tests using only SSR dataset or combined dataset (SSR + mtDNA) we obtained poor values of ESS (ESS <11). Therefore, it was not possible to use the SSR loci (alone or with mtDNA dataset) in IMa2 analysis because the runs did not converge and became stationary. Thus, we implemented this analysis only using mtDNA dataset.

Finally, we tested for evidence of recent population bottleneck events in the SSR dataset in BOTTLENECK v. 1.2.0.2 [40]. The run for each population (eastern and western groups) was based on the stepwise mutation model (SMM) and the significance was evaluated by Wilcoxon’s test with 10,000 replications. A significant number of loci with heterozygosity excess is expected in bottlenecked populations, while a heterozygosity deficit is expected in expanding populations [40].

### Testing Alternative Historical Scenarios by Approximate Bayesian Computation

**Fig 2:**
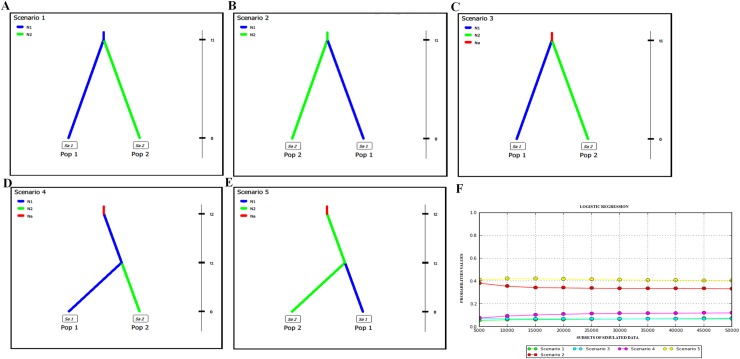
Graphic representation of the five scenarios tested for the eastern (pop1) and western groups (pop2) in DIYABC. In scenario 1 (A)—at t1, the eastern population group gives rise to the western group; scenario 2 (B)—at t1, the western group gives rise to the eastern group; scenario 3 (C)—at t1, an ancestral population splits and gives rise to both groups; scenario 4 (D)—at t2, an ancestral population gives rise to the eastern group, and at t1, the eastern group gives rise to the western group; scenario 5 (E)—at t2, an ancestral population gives rise to the western group, and at t1, the western group gives rise to the eastern group. The logistic regressions of the posterior probabilities for each scenario are shown in “F”.

To evaluate the fit of the different historical scenarios to the observed data for the *P*. *rustica* populations, we implemented the Approximate Bayesian Computation procedure (ABC) [41] available in DIYABC v2.1 [42]. We assumed the existence of two groups of populations (eastern and western of São Francisco River), based on our population structure tests (see Table 2). We compared competing hypotheses on the origin and dispersal of the *P*. *rustica* populations by investigating five potential historical scenarios: scenario 1—at t1, the eastern population group gives rise to the western group; scenario 2—at t1, the western group gives rise to the eastern group; scenario 3—at t1, an ancestral population splits and gives rise to both groups; scenario 4—at t2, an ancestral population gives rise to the eastern group, and at t1, the eastern group gives rise to the western group; scenario 5—at t2, an ancestral population gives rise to the western group, and at t1, the western group gives rise to the eastern group (see Fig 2). We estimated the posterior probability of these five putative scenarios for *P*. *rustica* using the mtDNA (all concatenated data) and SSR datasets both separately and together. For both datasets, the priors were set at a uniform distribution, with a generation time of one year. For the mtDNA dataset, the simulations were run for four million iterations, with a different set of summary statistics being generated for each population (the number of haplotypes and segregating sites) and between populations (the number of haplotypes, FST and the mean pairwise differences, w), with the mutation rate being left on the default setting. For the SSR dataset, the simulations were also run for four million iterations with summary statistics being generated for: (1) the number of alleles, (2) mean genetic diversity, (3) mean size variance, (4) FST, (5) the classification index and (6) the (dμ)^2^ distance. Once again, the mutation rate was left on the default setting. All other settings were configured as default settings of DIYABC for the SSR and mtDNA datasets. The posterior probability of each scenario was calculated by logistic regression, considering between 500 and 50,000 datasets that were closest to the observed values. The scenario that best explained the data was used to estimate the origin and dispersal of *P*. *rustica*.

**Table 2:** Genetic diversity estimates for the eight microsatellite loci and four concatenated mitochondrial gene regions (12S, 16S, COI and COI-COII) of the *Partamona rustica* populations and for the two population groups (Ar, allelic richness; He, expected heterozygosity; S, number of polymorphic sites; h, number of haplotypes; π, nucleotide diversity; Hd, haplotype diversity).

	Localities	Microsatellites		mtDNA (concatenated)			
		Ar	He	S	h	π	Hd
1	B. Vista do Tupim	2.041	0.242	0	1	-	-
2	Contendas do Sincorá	1.785	0.222	1	2	0.00018 (±0.00006)	0.409 (±0.133)
3	Iramaia	2.100	0.251	1	2	0.00024 (±0.00002)	0.533 (±0.052)
4	Ituaçu	2.995	0.383	1	2	0.00008 (±0.00006)	0.182 (±0.144)
5	Macaúbas	2.946	0.379	2	3	0.00029 (±0.00009)	0.582 (±0.142)
6	Milagres	1.961	0.243	3	5	0.00048 (±0.00005)	0.758 (±0.050)
7	Manoel Vitorino	2.475	0.293	0	1	-	-
8	Ruy Barbosa	1.849	0.270	1	2	0.00013 (±0.00007)	0.303 (±0.147)
9	Tanque Novo	3.076	0.354	4	6	0.00070 (±0.00009)	0.835 (±0.070)
10	Cocos	3.583	0.505	4	5	0.00066 (±0.00012)	0.800 (±0.100)
11	Cônego Marinho	3.621	0.473	3	4	0.00045 (±0.00009)	0.778 (±0.091)
***Groups***							
	Eastern (1–9)	2.359	0.288	15	22	0.00116 (±0.00005)	0.930 (±0.008)
	Western (10–11)	3.602	0.489	7	8	0.00085 (±0.00011)	0.879 (±0.040)
***All populations***		2.585	0.328	22	30	0.00146 (±0.00007)	0.946 (±0.006)

### Historical Climate Modelling

Once a contemporary model (i.e., 1950–2000) of the potential distribution of *P*. *rustica* was generated, we projected the model onto past climatic conditions to predict the potential distribution of the species during three distinct periods of the late Quaternary: the Last Interglacial period (LIG, 120 thousand years ago; or kya), the Last Glacial Maximum (LGM, 21 kya), and the Mid-Holocene (M-H, 6 kya). We modelled potential distributions using MaxEnt 3.3.3k [43, 44] based on the combined data for 20 localities, including the 11 localities sampled in the present study (Table 1) and a further nine sites recorded in the literature (S3 Table), with 19 bioclimatic environmental descriptors available in the WorldClim database (www.worldclim.org). Because the collinearity of variables can result in the overfitting of the model [45], we omitted correlated variables through a multivariate analysis (S1 Text).

We modelled the potential paleo-distribution of the species during the LGM and M-H periods using downscaled data derived from two Global Climate Models (GCMs) commonly used in recent analyses [46, 47], the CCSM4 and MIROC-ESM models, with a spatial resolution of 2.5 minutes. To estimate the areas that putatively remained suitable for the species throughout the Late Quaternary, we applied a threshold based on the minimum training presence (i.e., the value of the lowest contemporary logistic prediction) to transform continuous suitability outputs into presence/absence raster files. The areas of stability were then estimated from the intersection of all the projections (CCSM4 + MIROC-ESM) and separately, on a downscaled GCM. For further information, see S1 Text.

## Results

### Genetic Diversity

We obtained a total of 2,260 bp for the four mtDNA gene regions (Table 2). No indels were found in any of these regions. Nucleotide diversity (π) for the concatenated dataset was 0.00146 (±0.00007) and haplotype (h) diversity was 0.946 (±0.006) (Table 2).

Microsatellite loci revealed between two and 17 alleles. The allelic richness (standardised to a minimum sample size of eight) of the polymorphic loci ranged from 1.785 to 3.621, and the mean gene diversity (He—expected heterozygosity) was 0.328, ranging from 0.222 to 0.505 (Table 2). The genetic diversity of the western group was higher overall than that of the eastern group (see Table 2). Significant departures from HWE were found for the Mbi232 locus at ITU and Phel-1 at CMA, although no pairwise linkage disequilibrium was detected in any case. Finally, null alleles were detected only at BVT (Phel-1) and ITU (Phel-2 and Mbi232), and no evidence was detected of large allele dropout or scoring errors due to stuttering.

### Population Structure

We used different approaches to estimate the degree of population differentiation. Our haplotype network, based on the four mitochondrial gene regions, revealed the presence of two groups separated by two mutations, the first being restricted to the west of the São Francisco River Valley (SFRV), with eight haplotypes at COC and CMA localities, and the second in the east of the SFRV, represented by all the other nine populations and 22 haplotypes (Fig 1B).

The evolutionary model selected for the BI based on the AIC, including the outgroups, was TIM2 + I + G for COI, TIM3 + I for COII, TrN for 12S and TIM2 + I for 16S. The topology based on both methods of phylogenetic reconstruction was similar, showing at least two groups (eastern and western groups). In this sense, the Fig 1C shows the topology based on the BI, which showed considerable branches support for eastern group (posterior probability [PP] of 0.9), while western cluster is best regarded as a paraphyletic group. On the other hand, the ML showed poor value of support between these groups (see Fig 1C).

**Fig 3:**
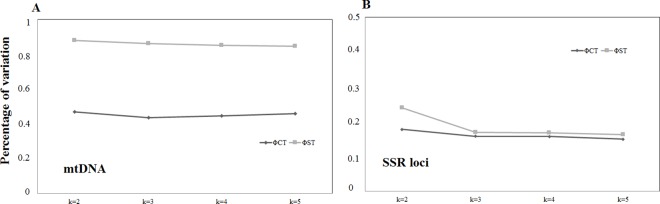
Spatial Analysis of Molecular Variance (SAMOVA) assessed for grouping schemes 2–5 of the *P*. *rustica* populations from the 11 localities based on the mitochondrial (A) and microsatellites (B) datasets, using estimates of fixation indices (Φ_CT_ and Φ_ST_). All *p*-values were significant (*p* < 0.005).

The AMOVA-based K-means cluster analysis of the SSR dataset indicated these two groups as the best clusters (S4 Table). The SAMOVA also confirmed the existence of the two groups (Table 3 and Fig 3) with moderate differentiation between the groups, based on both the mtDNA and SSR datasets (Φ_CT_ values in Table 3).

**Table 3:** Analysis of Molecular Variance (AMOVA) for three hierarchical levels, testing the differentiation between the *P*. *rustica* groups. The analysis was run using the four concatenated mtDNA genes and eight SSR loci.

Dataset	Source of variation	d.f	Variation (%)	Φ	*p*-Value
**mtDNA**	Between groups	1	46.88	Φ_CT_ = 0.468	<0.0001
	Among localities within groups	9	41.13	Φ_ST_ = 0.774	
	Within localities	134	11.99		
	**Total**	144			
**SSR**	Between groups	1	21.87	Φ_CT_ = 0.218	<0.0001
	Among localities within groups	9	2.85	Φ_ST_ = 0.247	
	Within localities	279	75.27		
	**Total**	289			

Whilst the Mantel test did not detect any significant correlation between genetic and geographic distances in the mtDNA data (r = -0.087, *p* = 0.627), it revealed a significant positive correlation between genetic and geographic distances for SSR dataset (r = 0.882, *p* < 0.001), indicating isolation by distance among the populations.

### Divergence Times and Migration

**Fig 4:**
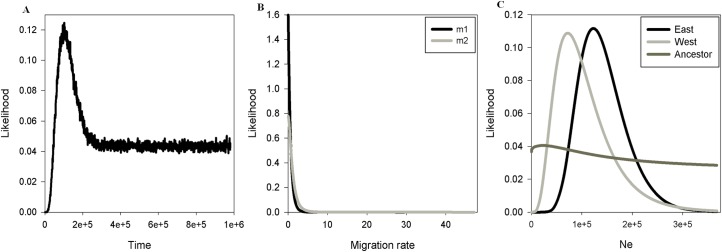
The IMa2 analysis of the *P*. *rustica* population groups from the west and east of the SFRV using the COI and the COI-COII gene fragment. In “A” Posterior probability distributions of the divergence time (t) between the two population groups is shown in years; in “B” Posterior probability distribution of migration (m) from west to east (m_west > east_) and east to west (m_east > west_); in “C” Posterior probability distribution of the Ne (effective population size), given as the number of individuals.

The estimate of the divergence time between the two groups of *P*. *rustica* by the IMa2 method indicated that the split occurred in the late Pleistocene at *ca*. 102 kya (HPD 95%: 45.588–979.902 kya). The isolation with migration model also revealed low migration rates between groups (Table 4 and Fig 4). The IMa2 estimates of the effective population size (Ne) showed that the peak of the probability distribution is found in the eastern population, followed by the western and ancestral populations.

**Table 4:** Estimates of demographic parameters by the IMa2 model for the *P*. *rustica* mtDNA dataset.

	t	m_west > east_	m_east > west_	θ_eastern_	θ_western_	θ_ancestral_
Highest point	102,451	0.01087	0.02573	122,610	71,875	22,610
Lower HPD of 95%	45,588*	0	0	59,007	18,934	0*
Upper HPD of 95%	979,902*	1.847	3.284	237,684	220,404	345,772*

HPD: High posterior density

t: Splitting time in years

θ: Effective population sizes of west, east and the ancestral population.

m: Migration rate in coalescent time.

*: HPD values with high precision and influenced by priors, because the likelihood distribution was flat at the end and did not touch zero.

### Population History by ABC

The SSR dataset was omitted from the ABC analyses because it did not adjust to any of the models tested, neither alone or together with mtDNA dataset. The ABC analysis for the mtDNA dataset identified scenario 5 as the origin and dispersal scenario with the highest posterior probability, followed by the scenario 2 (Fig 2). Both scenarios points to a western origin with a recent origin of eastern populations. In scenario 5, the ancestral originating western group at *ca*. 258 ka (HPD 95%: 7.0–476 ka) and western group then diverged to form the eastern group at *ca*. 13 ka (HPD 95%: 10–26 ka). In scenario 2, western group gave rise to the eastern group at *ca*. 14 Ka (HPD 95%: 10,500–34,300) (Fig 2). There is overlapping of confidence intervals between the posterior probabilities of the scenario 5 (P_S5_ = 0.4114, 0.3383–0.4845) and scenario 2 (P_S2_ = 0.3819, 0.2986–0.4651), therefore, we can consider both scenarios as probable.

### Historical Demography

**Fig 5:**
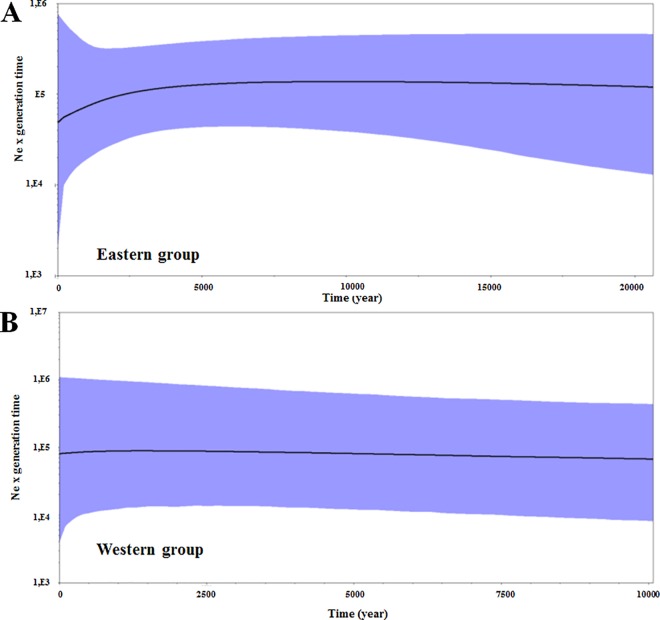
Coalescent Bayesian Skyline Plot (BSP) used to infer the demographic history of *P*. *rustica* populations to the east and west of the SFRV. The dotted horizontal line shows the median estimate of the BSP and the blue area shows the upper and lower 95% highest posterior density limits.

The BSP did not reveal any marked fluctuations in the effective size (Ne) of the population in either group, although there was a slight reduction in the median Ne values at 5 kya (Fig 5), although this was accompanied by an increase in confidence intervals. Similarly, no evidence was found of any recent population bottlenecks or demographic expansion in the SSR dataset of the western group (Wilcoxon’s test—heterozygosity deficit, *p* = 0.1250; heterozygosity excess, *p* = 0.9023). On the other hand, we found a non-significant heterozygosity excess (Wilcoxon: *p* = 1.0000) and a significant heterozygosity deficit (*p* = 0.0019) in the eastern group.

### Historical Distribution Patterns

**Fig 6:**
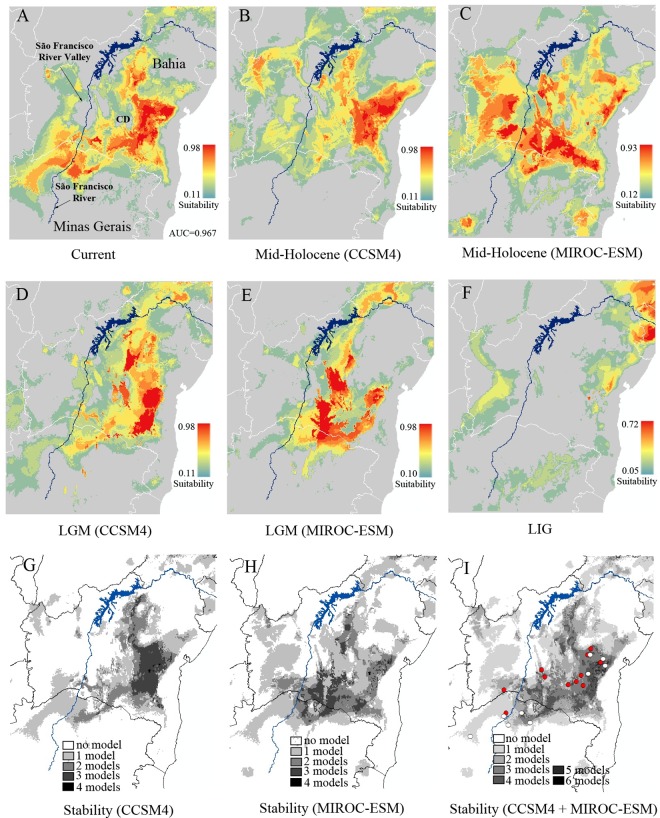
Ecological niche modelling showing the potential geographical distribution of *P*. *rustica* in different periods and stability models. In “A” Current, “B” and “C” Mid-Holocene, “D” and “E” Last Glacial Maximum (LGM) and “F” Last Interglacial period (LIG). The stability models (G to I) refer to the overlap of the potential distribution maps using two Global Climate Models combined (I) and each GCM separately (G and H). In “I”, the red points represent the localities sampled in the present study and white points represent other localities obtained from collections and museums. The legends indicate the probability of suitable conditions for the species. Abbreviations: CD = *Chapada Diamantina*; AUC = area under the curve.

Our ENM (Ecological Niche Model) analysis accurately predicted the current distribution of *P*. *rustica*, with an AUC of 0.96. During the LIG, few suitable areas were observed within the current range of *P*. *rustica* (Fig 6F), whereas a higher degree of suitability was observed during the M-H (Fig 6B and 6C), and a slightly lower one during the LGM (Fig 6D and 6E). All the models indicated climate stability to the east of the SFRV, although alternative outcomes were observed in the simulations for the western group (Fig 6). Considering that the downscaled Global Climate Models (GCM), i.e. CCSM4 and MIROC-ESM, are generated by different processes that naturally produce different outcomes, we present the results separately (Fig 6G and 6H), before combining the simulations in a consensus model (Fig 6I). Zones of stability were defined here as areas in which at least three models indicated the potential local occurrence of *P*. *rustica* during the historical period analysed (Fig 6G, 6H and 6I).

Despite some differences among the models, a continuum of putatively stable areas for *P*. *rustica* was identified to the east of the SFRV in all models. This zone covers central Bahia, including the *Chapada Diamantina* hills, and northern state of Minas Gerais (Fig 6I). Almost all the eastern populations are found in this potentially stable area, which is characterised by a high degree of historical suitability, whereas the western group is located within an area of much lower stability. This analysis also pointed to climatic instability in the SFRV and north-eastern Bahia (Fig 6I).

## Discussion

### Phylogeographic Structuring and Evolutionary History

Overall, our population structure analyses (haplotype network, phylogenetic reconstruction, K-means and SAMOVA) of both mtDNA and SSR datasets were consistent in reveal two groups of populations in *P*. *rustica*, one distributed to the east of the SFRV, and the other to the west. The IMa2 approach indicated that the two groups diverged in the late Pleistocene. While the haplotype network and ENM suggest that *P*. *rustica* lineages origin occurred in areas to the east of the SFRV, given the shared haplotype in greater frequency (H10 in Fig 1B) and the greater potential areas in the eastern part of the distribution, respectively, the ABC analysis proposed a historical process in which the *P*. *rustica* populations diversified from an ancestral population that initially gave rise to the western group (found in north-western Minas Gerais), which then dispersed to the east of the SFRV, where the species probably colonised central and south-western Bahia (scenario 5 in Fig 2E). Another probable hypothesis is that *P*. *rustica* populations diverged from western group, which then dispersed to the east of the SFRV, as showed by the scenario 2 (Fig 2B). Therefore, both scenarios have shown the western group as ancestral of the eastern group, which also is supported by phylogenetic inferences (Fig 1C). These findings probably justify the greater genetic diversity found in the SSR data for the western group in comparison with the eastern group, despite smaller sample size available for the former group. This result agrees with other study, which suggested that *P*. *rustica* is endemic to the region that stretches from northern Minas Gerais through the Espinhaço hills to south-western Bahia [48]. Therefore, here we considered the western group as ancestral of eastern group, since the ABC approach and phylogenetic reconstruction by BI and ML inferences are more robust methods to infer relationships among lineages.

Our analyses indicated that the two *P*. *rustica* groups exhibit reduced levels of migration between them. This reflects the moderate degree of differentiation between groups in both the mtDNA and the SSR dataset, which contrasts with the within-group variation especially at the SSR markers, and may be accounted for by the relatively recent evolutionary history of *P*. *rustica*. However, the divergence time indicated by the ABC approach was shorter than that suggested by the IMa2, which may be due to the migration between groups, which is not contemplated by the DIYABC, which assumes an absence of migration among populations after they have diverged [42].

While the present results reveal a moderate degree of differentiation between population groups of *P*. *rustica*, the probable gap of 200 Km between groups hampers the analysis of the potential role of the SFRV as a barrier to gene flow. Moreover, the SFRV region was determined to be an area of instability for the species (Fig 6G, 6H and 6I), which may have exerted an influence on sampling in this region. Alternatively, the differentiation between *P*. *rustica* population groups may be due to ecological differences (ecotone habitats in the west *vs*. the *Caatinga* in the east) and isolation by distance, as found in the SSR data, since this stingless bee might have low vagility [16, 49], or may be explained by fact that the species is limited to mountain areas (see Fig 1), being scarce in lowland areas, such as the SFRV.

### Isolation by Distance and Sex-Biased Dispersal

The Mantel test did not find evidence of isolation by distance in the mtDNA data, indicating that the differences found among populations cannot be accounted for by the physical distances among them. By contrast, strong evidence of isolation by distance was found in the SSR dataset. Similarly, while the mitochondrial genes indicated a high degree of differentiation between populations, only moderate differentiation was found in the SSR dataset (Table 3). These apparent disagreements are common in recently-diverged lineages and may be the result of long-term, male-biased dispersal, which results in genetic structuring in the mtDNA, but panmixia in the nuDNA (SSR) [50]. In this sense, new *Partamona* queens are phylopatric, remaining in their place of origin [51]. These new queens disperse no more than 300 meters from the maternal nest in the swarming process and workers demonstrate limited dispersal capacity [51, 49]. Moreover, like many species of Hymenoptera, stingless bees have a peculiar system of sex determination (complementary sex determination—*csd*), in which individuals that are heterozygous at the *csd* locus are females, while in the hemizygous condition they develop into haploid males [52, 53]. However, diploid homozygotes at the sex locus develop into diploid males, which are highly harmful to colonies and populations, since they may be sterile, have a low survival rate into adulthood or produce diploid sperm [53, 54, 55, 56]. Studies report the occurrence of diploid males in stingless bees [57, 58, 59] and other hymenopterans [60]. Given the *csd* system and the phylopatric queen, it is crucial for this insect to have a mechanism to prevent inbreeding and homozygosis at the *csd* locus to avoid the generation of diploid males.

Therefore, this disagreement between the isolation by distance tests, together with the observed population differentiation, can be explained by the phylopatric females and dispersing males in *P*. *rustica*, featuring a sex-biased dispersal pattern. So, the acquisition of new genes by populations should occur as a result of the dispersal behaviour of the males, as observed in others stingless bees [61, 62, 63], orchid bees (Euglossini) in the Neotropical region [64, 65, 66] and other hymenopterans [67]. Alternatively, these results may reflect conspicuous differences in the history of genetic markers within organisms, i.e., modes of inheritance and degree of ploidy [50].

### Historical Demography and Paleomodeling

The BSP yielded consistent results indicating that there were no marked changes in effective population size in either the western or eastern groups of *P*. *rustica* (Fig 5). While we found a significant heterozygosity deficit on SSR data in the eastern group, which might indicate a process of expansion, this signal may actually correspond to a false signal of recent demographic expansion induced by isolation by distance, asymmetric gene flow and the recent emergence of rare alleles through migration [68, 69], all scenarios that were observed during the present study. These results were confirmed by the paleomodeling, which showed a marked increase in the potential range of *P*. *rustica* from LIG to the present day, as well as areas of stability that harbour most of the known range of *P*. *rustica* (Fig 6I), including most of the localities sampled in this study. These areas may have acted as refuges for the species, in particular to the east of the SFRV. On the other hand, the results of our ENM also indicated few areas of stability in the western region, which may have been at least partly influenced by the relatively small number of records obtained from the western region in comparison with the eastern portion of the study area. Therefore, our results indicate that the inferences both from the genetic data analyses and from the spatial distribution modelling are compatible with historical demographic stability.

The refuge areas proposed here for *P*. *rustica* (Fig 6I) overlap the SDTF refuge proposed in other study for the southern portion of the Southern Backlands Depression (*Depressão Sul-Sertaneja*) of Bahia [13]. Our results also point to the southern *Chapada Diamantina* and the SFRV as areas of instability for the species, as well as the north-eastern Bahia (Fig 6G, 6H and 6I), as observed in previous study to SDTF areas [13]. Our findings also confirm the potential current distribution proposed to *P*. *rustica* [16]. In addition, the potential current distribution of *C*. *cyphergaster* termite mounds, which are the main substrate used by *P*. *rustica* to build nests [16], covers the potential current distribution of *P*. *rustica* and indicated the *Cerrado* as an area of higher potential occurrence for *C*. *cyphergaster* and the *Chapada Diamantina* as an area of low potential occurrence for this species [70]. This may explain the absence of *P*. *rustica* in *Chapada Diamantina* [16]. Future phylogeographic studies involving both *P*. *rustica* and *C*. *cyphergaster* should be conducted to gain a better understanding of the phylogeography of *P*. *rustica*, given its close relationship with this termite.

The use of a single molecular marker, such as mtDNA, to infer demographic history, divergence time, the origin of a species, and its history of dispersal has certain limitations and potential biases [71], although the validity of a molecular marker depends primarily on the structure of populations being analysed. In this context, some characteristics of the stingless bees, such as limited long-distance migration [49], female phylopatry [51], and colonies composed primarily of females make the analysis of maternally-inherited mtDNA markers a highly suitable approach for studying the evolutionary history of these animals. In this case, the population history of the females is what probably determines the long-term reproductive success and survival of the population. Overall, then, while the exclusive analysis of mtDNA markers does not cover the entire demographic and evolutionary history of a species, it has proven to be a very useful tool for the study of closely-related populations [72, 73].

### Conservation Biogeography of Stingless Bees in Dry Forests

Understanding the history of the distribution of a species is essential for the development of effective conservation strategies, especially for species such as *P*. *rustica*, which has a restricted distribution and its populations are differentiated in two groups. In addition, bee hunters represent a serious threat to *P*. *rustica*, which has been overexploited for the collection of honey, pollen, and wax, with the result that nests are becoming rare or even absent in many areas, contrasting to its importance as a pollinator in *Caatinga* and *Cerrado* areas [16]. Deforestation and livestock farming have further contributed to the extinction of the species in some areas [16]. Due to the many threats in these areas, other studies involving stingless bees have also shown the need to conserve stingless bees in the *Caatinga* [16, 74] and *Cerrado* areas [75, 76]. Investigations involving organisms from dry forests are important, especially due to the fact that such organisms have been largely excluded from discussions on conservation [77].

While studies on the biogeography and phylogeography of the taxa found in dry forest environments are increasing, the role of Pleistocene glaciations and vicariant events in the diversification of this biota are still poorly investigated. These ecosystems have high levels of endemism [77], which have been threatened by ongoing anthropogenic impacts. For this reason, it is essential to understand the evolutionary processes that have shaped the genetic diversity of the dry forest biota in order to develop appropriate conservation strategies. Our results contribute to the interpretation of the biogeographic scenarios that arose in the *Caatinga* and *Cerrado* areas during the Pleistocene and reinforce the need for further, more detailed investigation of the dry environments of the Neotropical region.

## Supporting Information

S1 Table Primers used for PCR of mitochondrial genes amplifications. (DOCX)

S2 Table Repeat motifs, annealing temperatures (Ta) and each respective fluorophores of the microsatellite loci used for analysis of *P*. *rustica*. (DOCX)

S3 Table Records of occurrence of *P*. *rustica* obtained in Camargo and Moure’s collection used in ecological niche modeling. (DOCX)

S4 Table AMOVA-based K-means clustering by pseudo-f statistics [1]. The test indicates two groups (eastern and western–see Table 2).
(DOCX)

S1 Text Details of the methods used. (DOCX)
