# Supplementary material for: Phylogeography of Partamona rustica (Hymenoptera, Apidae), an Endemic Stingless Bee from the Neotropical Dry Forest Diagonal
Source: PLoS One. 2016 Oct 10;11(10):e0164441. doi: 10.1371/journal.pone.0164441 (PMC5056711; doi:10.1371/journal.pone.0164441)
Supplement: S1 Table — (DOCX) [file pone.0164441.s001.docx]

**S1 Table. Primers used for PCR of mitochondrial genes amplifications**.

| **Region** | **Size (in bp)** | **Primers** | **Reference** |
| --- | --- | --- | --- |
| **16S** | **569** | F: 5’-TATAGATAGAAACCAATCTG-3’ | [1] |
|  |  | R: 5’-CACCTGTTTATCAAAAACAT-3’ |  |
| **12S** | **412** | F: 5’-TACTATGTTACGACTTAT-3’ | [2] |
|  |  | R: 5’-AAACTAGGATTAGATACCC -3’ |  |
| **COI** | **629** | F: 5’-GGAGATCCAATTCTTTATCAAC-3’ | [3] |
|  |  | R: 5’-GATATTAATCCTAAAAAATGTTGAGG-3’ | [4] |
| **COI-COII** | **650** | F: 5’-TCTATACCACGACGTTATTC-3’ | [1] |
|  |  | R: 5’-GATCAATATCATTGATGACC-3’ |  |

**References**

1. Hall HG, Smith DR. Distinguishing African and European honeybee matrillines using amplified mitochondrial DNA. Proc Natl Acad Sci USA. 1991; 88: 4548-4552.
2. Simon C, Frati F, Beckenbach A, Crespi B, Liu H, Flook P. Evolution, weighting, and phylogenetic utility of mitochondrial gene sequences and a compilation of conserved polymerase chain reactions primers. Ann Entomol Soc Am. 1994; 87: 6.
3. Afonso J. Origem das linhagens mitocondriais nas abelhas africanizadas (Apis mellifera L.) do Brasil. MSc Thesis, Universidade Federal de São Carlos, São Carlos. 2012.
4. Dick CW, Roubik DW, Gruber KF, Bermingham E. Long-distance gene flow and cross-Andean dispersal of lowland rainforest bees (Apidae: Euglossini) revealed by comparative mitochondrial DNA phylogeography. Mol. Ecol. 2004; 13: 3775-3785.
