# Supplementary material for: Phylogeography of Partamona rustica (Hymenoptera, Apidae), an Endemic Stingless Bee from the Neotropical Dry Forest Diagonal
Source: PLoS One. 2016 Oct 10;11(10):e0164441. doi: 10.1371/journal.pone.0164441 (PMC5056711; doi:10.1371/journal.pone.0164441)
Supplement: S2 Table — (DOCX) [file pone.0164441.s002.docx]

**S2 Table. Repeat motifs, annealing temperatures (Ta) and each respective fluorophores of the microsatellite loci used for analysis of *P. rustica*.**

| **Locus** | **Motif** | **Size** | **Ta (ºC)** | **Fluorophore** | **Primer** |  |
| --- | --- | --- | --- | --- | --- | --- |
| **Phel-1** | AC | 217-247 | 55ºC | TET | F: 5’- TCGGCCGCTCATGGATAAGT- 3’ | |
|  |  |  |  |  | R: 5’ - TCAACGCCAGTCGAGAAGAGGATG - 3’ | |
| **Phel-2** | TC | 260-312 | 52ºC | TET | F: 5’- CGTTCAATTTACCGCACAA - 3’ | |
|  |  |  |  |  | R: 5’ - CCACGTATCCAGGCTTTTTA - 3’ | |
| **Phel-3** | CT | 99-101 | 55ºC | JOE | F: 5’ - GTCGCAATAGCAATAGG - 3’ | |
|  |  |  |  |  | R: 5’ - TGGTCGTCATCTGTTTT - 3’ | |
| **Phel-4** | GT | 246-254 | 62ºC | FAM | F: 5’ - AATAACACGCGCACCATCA - 3’ | |
|  |  |  |  |  | R: 5’ - ACACATACAGAAGAACGAAGAAAA - 3 | |
| **Phel-6** | CA | 174-180 | 52ºC | HEX | F: 5’ - TTGGCACGAAAAGAACA - 3’ | |
|  |  |  |  |  | R: 5’ - TTGAAAGCTGAAAAATCCA - 3’ | |
| **Phel-7** | TG | 282-298 | 48ºC | HEX | F: 5’ - TTACATAAGAGCAAAACT - 3’ | |
|  |  |  |  |  | R: 5’ - TCGAAAATGAAATAAATA - 3’ | |
| **Mbi-254** | AAG | 208-232 | 52ºC | FAM | F: 5’ - CAATCGTTGGAAGGGAAC - 3’ | |
|  |  |  |  |  | R: 5’ - GGACCTATACCCAAGTCCAT - 3’ | |
| **Mbi-232** | CTT | 130-178 | 48ºC | FAM | F: 5’ - TTTTTCTCTTAAATTTTCTTCT - 3’ | |
|  |  |  |  |  | R: 5’ - CTTACTCGACGACTTTATTT - 3’ | |
