# Supplementary material for: Phylogeography of Partamona rustica (Hymenoptera, Apidae), an Endemic Stingless Bee from the Neotropical Dry Forest Diagonal
Source: PLoS One. 2016 Oct 10;11(10):e0164441. doi: 10.1371/journal.pone.0164441 (PMC5056711; doi:10.1371/journal.pone.0164441)
Supplement: S3 Table — (DOCX) [file pone.0164441.s003.docx]

**S3 Table. Records of occurrence of *P. rustica* obtained in Camargo and Moure’s collection used in ecological niche modeling.**

| **Site** | **S** | **W** |
| --- | --- | --- |
| Bonfinópolis de Minas-MG | -16.441 | -46.110 |
| Lontra-MG | -15.897 | -44.294 |
| Nova Porteirinha-MG | -15.776 | -43.252 |
| Januária-MG | -15.470 | -44.512 |
| Jaíba-MG | -15.317 | -43.650 |
| Maracás-BA | -13.424 | -40.447 |
| Amargosa-BA | -13.052 | -39.631 |
| Itatim-BA | -12.723 | -39.728 |
| Itaberaba-BA | -12.512 | -40.351 |
