# Supplementary material for: Phylogeography of Partamona rustica (Hymenoptera, Apidae), an Endemic Stingless Bee from the Neotropical Dry Forest Diagonal
Source: PLoS One. 2016 Oct 10;11(10):e0164441. doi: 10.1371/journal.pone.0164441 (PMC5056711; doi:10.1371/journal.pone.0164441)
Supplement: S4 Table — The test indicates two groups (eastern and western–see Table 2). (DOCX) [file pone.0164441.s004.docx]

**S4 Table. AMOVA-based K-means clustering by pseudo-f statistics [1]. The test indicates two groups (eastern and western – see Table 2)**

| **numK** | **SS-total** | **SS-among** | **SS-within** | **r-squared** | **pseudo-f** | **Phi_ct** |
| --- | --- | --- | --- | --- | --- | --- |
| **1** | 472.63 | 0.00 | 116.91 | 0.000 | 0.000 | 0.000 |
| **2** | 472.63 | 66.53 | 406.10 | 0.569 | **11.887** | 0.383 |
| **3** | 472.63 | 82.83 | 389.80 | 0.709 | 9.725 | 0.281 |
| **4** | 472.63 | 90.81 | 381.83 | 0.777 | 8.118 | 0.221 |
| **5** | 472.63 | 97.72 | 374.91 | 0.836 | 7.641 | 0.228 |

1. Calinski R, Harabasz J. A dendrite method for cluster analysis. Commun Stat. 1974; 3: 1-27.
