# Supplementary material for: Phylogeography of Partamona rustica (Hymenoptera, Apidae), an Endemic Stingless Bee from the Neotropical Dry Forest Diagonal
Source: PLoS One. 2016 Oct 10;11(10):e0164441. doi: 10.1371/journal.pone.0164441 (PMC5056711; doi:10.1371/journal.pone.0164441)
Supplement: S1 Text — (DOCX) [file pone.0164441.s005.docx]

**S1 Text - Details of the methods used**

**Amplification of the mitochondrial DNA**

The PCR (25 μL) contained template DNA (50 ng), 1X of Taq buffer (Invitrogen), 250 μM of each dNTP, l.0 μM of each primer, 2.5 mM of MgCl_2_ and 1 U of Taq polymerase (Invitrogen). The PCR conditions were as follow: an initial denaturation step at 94ºC for 5 minutes, followed by 40 cycles of denaturation at 94°C for 30 s, annealing at 50°C (COI, COI-COII and 12S) or 48ºC (16S) for 20 s, and extension at 70°C for 1 min, with final extension at 70°C for 10 min. The PCR products were electrophoresed in agarose gel stained with Gel Red^TM^.

The amplified DNA was purified using illustra ExoProStar 1-Step kit (GE). The purified products were used as templates for sequencing in both forward and reverse directions. The DNA sequencing was carried out using a BigDye v 3.0 Dye Terminator Cycle Sequencing kit (Applied Biosystems, Inc., Carlsbad, CA, USA) following the manufacturer’s protocol, with the same primers used for the amplification. The sequences were analysed in an ABI 3730 XL automated sequencer (Applied Biosystems).

**Genetic structure analysis**

We used the AMOVA-based K-means clustering method [1] for the analysis of the populations groups. This procedure was implemented in kMeans v.1.1 (http://www.patrickmeirmans.com/software/) using the SSR dataset, following the settings recommended by the author of the program: a maximum number of clusters of 11; AMOVA, to calculate the distances; Pseudo-F to determine the optimal number of clusters; simulated annealing, as a clustering algorithm; 50,000 steps for the simulated annealing chain; and 10 repeats of the algorithm. We also used the Spatial Analysis of Molecular Variance (SAMOVA) to test for geographical groupings that are genetically homogeneous and maximally differentiated from each other, without making *a priori* assumptions on group assignments, using both mtDNA (all concatenated gene regions) and SSR datasets separately; this approach was implemented in SAMOVA 2.0 [2]. In this analysis, we tested for the existence of between two and five groups (k2 – k5), based on 1000 simulations of the annealing process in each case. We then compared the fixation indices (Φ_ST_ – structure among localities among groups; Φ_CT_–structure among groups) and percentage of variation between ks and chose the k with the highest levels of differentiation.

**Historic climate modelling**

As redundant variables may cause overfitting of the outputs of the modelling, we identified variables that were highly correlated with one another using the multivariate Principal Component Analysis (PCA), run in PAST [3]. We excluded those variables with the lowest percentage contribution to the final model of *P*. *rustica* distribution under current conditions. This is the only step that differs from [4], and it was applied after the assignment of the values of each bioclimatic variable to the *P. rustica* occurrence records using the “extract multi values to points” tool in ArcMap 10.1. Following this step, we constructed a correlation matrix in PAST to produce the scatter diagram resulting from the PCA. The redundant variables were identified from their respective contribution to the model, and the correlated variables with the lowest percentage contribution to the model were eliminated [5]. Based this approach, we removed two variables, BIO1 and BIO19. The final model was built after the exclusion of these variables, and the projections of past scenario were implemented using the same MaxEnt parameters described by [5] during the construction of the definitive model of climatic stability for *P*. *rustica*.

Ecological niche modelling was conducted based on the minimum area for the known range of occurrence of the species in order to minimise the influence of background noise on the model output [6] Anderson. The potential distribution map was edited in Arc-Gis 10.1 and we used DIVA-GIS 7.5.0 (<http://www.diva-gis.org/>) to define the overlap of the potential distribution maps generated for different periods and identify the putative areas of stability within the range of *P. rustica.*

**References**

1. Meirmans PG. AMOVA-based clustering of population genetic data. J Hered. 2012; 103: 744-750
2. Dupanloup I, Schneider S, Excoffier L. A simulated annealing approach to define the genetic structure of populations. Mol Ecol*.* 2002; 11: 2571-2581.
3. Hammer Ø, Harper DAT, Ryan. PD PAST: paleontological statistics software package for education and data analysis. Palaeontol Electron*.* 2001; 4: 1-9.
4. Carvalho AF, Del Lama MA. Predicting priority areas for conservation from historical climate modelling: stingless bees from Atlantic Forest hotspot as a case study. J Insect Conserv*.* 2015; 19: 581-587.
5. Miranda EA, Carvalho AF, Andrade-Silva ACR, Silva CI, Del Lama MA. Natural history and biogeography of *Partamona rustica*, an endemic bee in dry forests of Brazil. Insect Soc*.* 2015; 62: 255-263.
6. Anderson RP, Raza A. The effect of the extent of the study region on GIS models of species geographic distributions and estimates of niche evolution: preliminary tests with montane rodents (genus *Nephelomys*) in Venezuela. J Biogeog. 2010; 37: 1378-1393.
